# Newcastle Disease Virus Interaction in Targeted Therapy against Proliferation and Invasion Pathways of Glioblastoma Multiforme

**DOI:** 10.1155/2014/386470

**Published:** 2014-08-27

**Authors:** Jafri Malin Abdullah, Zulkifli Mustafa, Aini Ideris

**Affiliations:** ^1^Center for Neuroscience Services and Research, Universiti Sains Malaysia, Health Campus, 16150 Kubang Kerian, Kelantan, Malaysia; ^2^Department of Neurosciences, School of Medical Sciences, Universiti Sains Malaysia, Health Campus, 16150 Kubang Kerian, Kelantan, Malaysia; ^3^Department of Neurosciences, Hospital Universiti Sains Malaysia, Jalan Sultanah Zainab 2, Kubang Kerian, 16150 Kota Bharu, Kelantan, Malaysia; ^4^Faculty of Veterinary Medicine, Universiti Putra Malaysia, 43400 Serdang, Selangor, Malaysia

## Abstract

Glioblastoma multiforme (GBM), or grade IV glioma, is one of the most lethal forms of human brain cancer. Current bioscience has begun to depict more clearly the signalling pathways that are responsible for high-grade glioma initiation, migration, and invasion, opening the door for molecular-based targeted therapy. As such, the application of viruses such as Newcastle disease virus (NDV) as a novel biological bullet to specifically target aberrant signalling in GBM has brought new hope. The abnormal proliferation and aggressive invasion behaviour of GBM is reported to be associated with aberrant Rac1 protein signalling. NDV interacts with Rac1 upon viral entry, syncytium induction, and actin reorganization of the infected cell as part of the replication process. Ultimately, intracellular stress leads the infected glioma cell to undergo cell death. In this review, we describe the characteristics of malignant glioma and the aberrant genetics that drive its aggressive phenotype, and we focus on the use of oncolytic NDV in GBM-targeted therapy and the interaction of NDV in GBM signalling that leads to inhibition of GBM proliferation and invasion, and subsequently, cell death.

## 1. Introduction

Oncolytic viruses are viruses that selectively eradicate tumour cells without harming the normal surrounding tissues [1–3]. They are used to recognise and infect mutated cancerous cells, where they replicate and then release new virions that directly amplify the input dose. Newly produced virions can also spread and infect the adjacent cancerous cells. Consequently, infected cells often undergo pathological programmed cell death, known as apoptosis [4].

Grade IV glioma, or glioblastoma multiforme (GBM), is one of the most lethal forms of human brain cancer, despite multiple modern approaches that have been developed to combat the disease [5]. Current bioscience has now begun to depict more clearly the signalling pathways responsible for high-grade glioma initiation, migration, and invasion, thus opening the door for molecular-based targeted therapy [6]. Targeted therapy is a therapeutic approach that uses a specific molecule inhibitor or activator to hinder or reboot the aberrant signalling observed in cancerous cells.

The application of viruses as a novel biological bullet to specifically target aberrant signalling in GBM has brought new hope. Newcastle disease virus (NDV), a chicken pathogen that exhibits selective oncolytic properties, is one of the most intensively studied oncolytic viruses, affecting many types of human cancer [7, 10, 11]. We previously presented the therapeutic potential of NDV to induce apoptosis in GBM cell cultures and induce GBM regression in in vivo and ex vivo models [7, 12]. As a mode of therapy, oncolytic NDV has been shown to be a potent and safe anticancer agent for treating human brain cancer [2]. As such, in the present review, we describe the therapeutic potential pathways associated with oncolytic NDV tropism in human GBM, which display the natural selectivity of NDV towards GBM and the interaction of NDV in GBM proliferation and invasion signalling.

## 2. Malignant Brain Cancer

Brain cancer is a mixed group of neoplasms that originate in intracranial tissue and meninges and display multiple levels of malignancy [13, 14]. Glial cancers, or glioma, the most common types of primary brain cancer, are derived from mutated glial cells and consist of astrocytes, oligodendroglial cells, and ependymal cells. As a form of cancer, gliomas are defined as pathological tumours that display histological, immunohistological, and ultrastructural evidence of glial differentiation [8, 14]. Among gliomas, GBM brain cancer is the most dangerous type of brain tumours, and no cure has been identified.

Malignant brain cancer is characterised by highly invasive multifocal growth, histologic and genetic heterogeneity, and local relapse [15, 16]. The complex characteristics of GBM explain its resistance to current therapeutic intervention.

As indicated by the name glioblastoma multiforme, this type of tumour is grossly multiformed and often haemorrhaging, with necrotic regions. It is also multiformed microscopically, with pleomorphic nuclei and cells, microvascular proliferation, and regions of pseudopalisading necrosis [15, 17, 18].

Another hallmark of high-grade brain cancer is its invasive nature. Due to the massive growth of the brain cancer focus, peripheral cancerous cells invade the adjacent brain parenchyma, and the core of the tumour becomes necrotic, forming a region in which tumour cells, oedema, and normal tissue coexist, making it difficult to estimate the tumour margin to ensure complete therapeutic removal [5, 19]. The tumour is also surrounded by a penumbra of invasive tumour cells that are detectable several centimetres away from the main tumour mass. These locally invasive glioma cells, which are often found at the margins of the tumour resection, are the most common sites of malignant glioma recurrence [20].

Brain cancers are relatively rare compared to other tumours, with an estimated 25,000 new patients diagnosed in North America in 2009 [21]. The majority of these patients have gliomas (>15,000), and of those, approximately 70% are GBM (WHO grade IV), 15% are anaplastic astrocytomas (WHO grade III), and the remainder are low-grade gliomas [2, 5]. In Malaysia, the incidence of cancers of the brain and nervous system, as reported by the Malaysian Cancer Registry, was 3.3 per 100,000 persons in 2006. This number reflects an increase from 2.4 per 100,000 in 2003, and the frequency is higher in males. Brain cancer is currently reported as the third most common paediatric cancer in Malaysia [7, 22, 23].

Despite the impressive advances in imaging, surgery, and therapy methodologies over the past 25 years, the median survival rate of GBM patients remains only 12–15 months [2]; thus, an urgent, proficient solution is needed.

## 3. GBM Genetic Aberrations

Generally, a cancer consists of mutated cells that divide or survive when, instead, they should undergo cell cycle arrest or apoptosis cell death due to internal aberrations. Thus, due to several genetic abnormalities, most cancers, including GBM, remain alive and can form tumours. The discovery that cancer is an aberrant genetic disease, arising when defects occur in genes involved in cell death and growth regulatory processes, has revolutionised our understanding of tumorigenesis [14, 24].

Several genetic aberrations in the genes governing cell cycle control and growth factor signalling pathways have been well described in human brain cancers [6, 25]. Genes that are mutated or amplified to lead to the enhancement of cellular growth are referred to as oncogenes. Glioma oncogenes have provided new insights into tumorigenesis, and therefore, the deregulated cell signalling pathways that have been identified are now becoming the focus of specific molecular targeted therapies [26].

**Figure 1:**
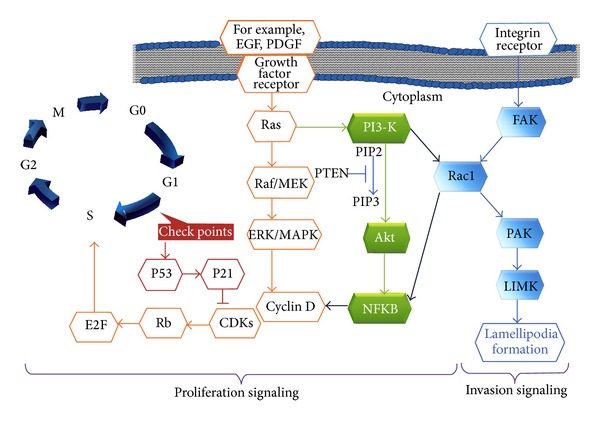
Genomic aberration of the proliferative and invasive pathways of glioma signalling. The extrinsic activation of growth factor receptors triggers the major signal transduction crossroad Ras-GTP, which conveys the message into the Raf-MAPK-ERK pathway or the PI3K-AKT or PI3K-Rho GTPase Rac1 pathway and leads the cell through the G1 restriction points of cell cycle. This signalling activation promotes an aberrant cell cycle that continuously produces mutated cells and promotes invasion signalling, resulting in an aggressive phenotype [6–8]. The control over cell division at checkpoint 1 is normally maintained by p53, a tumour suppressor that also contributes to DNA repair and cell death pathways.

Several deregulated signalling pathways have been described in GBM, mainly in proliferation signalling, including the MEK/ERK, PI3K/Akt, and PLC/PKC pathways. The deregulation of these signalling pathways is driven by the mutation, overexpression, or amplification of multiple genes, such as epidermal growth factor (EGFR), platelet-derived growth factor (PDGF), phosphatase and tensin homologue (PTEN), p53, retinoblastoma (Rb), and mammalian target of rapamycin (mTOR) [14, 16, 27]. A summary of the signalling regulations is shown in Figure 1.

Specifically, EGFR and the loss of chromosome 10 are the primary alterations found in GBM. EGFR amplification is found in nearly 92% of astrocytomas. By contrast, 62% of grade IV gliomas show an increased expression of EGFRvIII, a constitutively active mutant receptor. The complete loss of chromosome 10 has been reported in 70% of primary GBMs, whereas the other 30% display an aberrant tumour suppressor gene p53 [6, 25, 28].

Regardless of the cellular receptor or ligand status, up to 100% of GBMs show the activation of Ras, and nearly 70% show activated Akt. The loss of the tumour suppressor gene PTEN on chromosome 10, which normally represses Akt activation, is also typically observed.

As summarised in Figure 1, proliferation signalling of GBM is initiated after appropriate mitogenic signals, such as EGF or PDGF activation. Activated EGF receptor (EGFR) or PDGF receptor (PDGFR) triggers the synthesis of cyclin D, which enables cyclin-dependent kinase (CDK) via the Raf/ERK/MAPK or PI3K/Akt pathway. Active CDKs, such as CDK4, further phosphorylate and inactivate the tumour suppressor protein Rb. In turn, Rb is unbound from E2F, allowing this transcription factor to lead the cell through the G1 restriction point [18, 29, 30], subsequently allowing the cell to undergo genomic synthesis and mitosis to produce new cells. Furthermore, Rb is a major regulator of cell cycle progression; the mutational inactivation of Rb leads to unscheduled cell cycle entry, and Rb mutation is found in approximately 25% of GBMs [6, 8, 14].

In an overexpressing Rac1 NIH3T3 mutant cell, cyclin D transcription can be activated directly by the downstream of Rac1 (Figure 1) via the nuclear factor kappa-light-chain-enhancer of activated B cells (NFKB) [31, 32] to promote cell cycle progression [33, 34]. Aberrant cell proliferation associated with the constitutive activation of NFKB in response to PDGF overexpression has also been reported in gliomas. This NFKB activation is mediated via the PI3K pathway in association with PTEN inactivation [6, 14].

Another concerning feature of GBM cells is their ability to invade the normal brain parenchyma individually. This ability is achieved through the dual signalling of proliferation and invasion pathways via PI3K/Rac1 signalling (Figure 1) [9, 14, 33], which maintains tumorigenic cell survival.

Due to this variability, which indicates that gliomas comprise multiple diseases, unique and different therapeutic tools are required [24, 35], which has driven the development of targeted therapies. The overexpression of these genes provides an opportunity for oncolytic viruses such as oncolytic NDV, which require the Rac1 protein in their replications in human cancer cells [36].

## 4. Oncolytic NDV

NDV is a highly contagious pathogen that affects avian species and causes severe economic losses to the poultry industry worldwide. NDV outbreaks were first reported in poultry from Java, Indonesia, followed by Newcastle-upon-Tyne in 1926 [11, 37]. Eighteen NDV strains from four lineages were later identified and classified as velogenic, mesogenic, and lentogenic according to their pathotypes [38, 39]. NDV is classified as a member of the Paramyxoviridae family of the Mononegavirales superfamily, in the* Avulavirus* genus [37].

The NDV genome consists of 15 kb pairs of nonsegmented, single-stranded RNA, which code for six main structural proteins. These genes, nucleocapsid (NP), phosphorylation (P), matrix (M), fusion (F), hemagglutinin-neuraminidase (HN), and RNA-dependent RNA polymerase (L) proteins, are found in a 3′ NP-P-M-F-HN-L 5′ arrangement [40, 41].

In researching human brain cancer, preclinical studies of oncolytic viruses in glioma emerged in the 1990s, when the first attenuated herpes simplex viruses (HSVs) and adenoviruses were used, followed by oncolytic reovirus. To date, four viruses have completed the phase 1 clinical trials: herpes simplex virus (strains HSV-1, HSV-1716, and HSV-G207), Newcastle disease virus (strains MTH-68/H and NDV-HUJ), adenovirus (Onyx-015), and reovirus. As a result of the trials, the viruses were declared safe to be injected directly into the brain, and no maximum tolerated dose (MTD) was reached. Some antiglioma activities were also observed. NDV showed the most promising benefits, as six patients exhibited tumour regression and three patients exhibited long-term survival [2].

The lentogenic NDV strain OV001/HUJ has been used in the treatment of patients with stage IV brain cancer. In the third stage of phase I/II clinical testing, the NDV-HUJ strain was intravenously administered in two parts to patients with primary GBM. In the first part, escalation steps at doses of 0.1, 0.32, 0.93, 5.9, and 11 BIU of NDV-HUJ were given in one cycle of five consecutive daily doses, followed by three additional cycles of 55 BIU. In the second part, maintenance doses consisting of two doses of 11 BIU weekly were given. The MTD was not reached. One patient maintained a near complete response 30 weeks after the start of dosing, and a second patient maintained stable disease for 26 weeks [42].

Several strains of NDV infection are known to induce multicascade, self-suicide apoptosis in many human neoplastic cells [4, 12, 43].

## 5. Natural Selectivity of Oncolytic NDV towards GBM

It is well known that mutations in multiple genes promote tumour evolution and contribute to a malignant phenotype [6, 44]. Features of transformed cells, including altered receptor expression, defective signalling pathways, oncogene activation, and increased cell cycling, have been shown to augment the capacity of viruses to replicate within cancerous cells [45].

In normal brain cells responding to viral infections, microglia and astrocytes respond to foreign nucleic acids, leading to the stimulation of the pattern-recognition receptors (PRRs), such as TLR-3, TLR-7, and TLR-9. The activation of PRRs subsequently activates type 1 interferon (IFN) [2], which further binds and activates the Janus kinases JAK1 and TYK2, which in turn phosphorylate the activators for STAT1 and STAT2 transcription. The STAT proteins then heterodimerize and form a complex with IRF9. This complex, known as ISGF3, further provides DNA recognition and simultaneously produces the IFN-stimulated genes (IGSs) that create the antiviral state in the target cells and block viral replication [11, 46]. This is particularly important because the normal IFN mechanism prevents oncolytic virus amplification within normal brain parenchyma.

In the study of Miyakoshi et al. [47], the activation of oncogenes in human cancer increased the activation of protein kinases, leading to interferon synthesis and the inhibition of tumorigenesis. In glioma, however, the antitumour IFN response is impaired by glioma-derivative immunosuppressing factors such as TGF-b, IL-10, prostaglandin E2, and gangliosides. TGF-b, the most prominent immune suppressor, plays a major role in glioma biology, where it is often overexpressed and has become a hallmark of gliomas [2].

IFN*β* is the principle antiviral factor secreted by infected cells in response to NDV infection. Therefore, IFN-defective tumour cells provide a greater opportunity than normal cells for NDV to replicate effectively. Thus, this replication-competent virus-selective mechanism is associated with the defect of the host IFN [10, 46].

As NDV is very sensitive to IFN, its replication is inhibited in IFN-competent normal cells, but not in transformed cells that fail to develop an appropriate antiviral state [48]. However, it has been reported that many transformed cells that do not show deficiencies in IFN signalling are still selectively destroyed [48]. Consistent with this finding, melanoma cells with a functional IFN system were infected with NDV-HUJ, indicating that IFN is not solely involved in NDV-induced oncolysis [49]. Thus, selective cellular catastrophe by NDV seems to vary according to cell type and NDV strain pathotype.

Nevertheless, NDV has been used in immunotherapy to trigger IFN signalling against transformed cells [40]. Previous reports have reported that NDV infection was a thousandfold more efficient in Ras-transformed cells [36, 50, 51]. Consistent with those findings, the aberrant signalling of GBM, as discussed above, indicated that more than 50% of GBMs were identified with high Ras expression and EGFR overexpression [6, 52, 53], leading to increased cell proliferation, especially in the primary GBMs [14]. This result might explain why NDV targets cancerous cells more efficiently than normal cells.

It would be of great interest to address the recent report that placed new interest on the Ras downstream protein Rac1. Puhlmann et al. [36] identified Rac1 as a protein with activity that is critical for both oncolytic virus sensitivity and the autonomous growth behaviour of Ras-transformed skin carcinoma cells.

## 6. Rac1 Signalling in the Proliferation and Invasion of GBM

Rac1, a Ras-related C3 botulism toxin substrate 1, is a member of the monomeric G-protein Rho GTPases. In proliferation signalling, this protein is involved in the regulation of gene transcription and G1 cell cycle progression [14, 29, 33, 54]. Gjoerup et al. [55] reported that in an embryonic mouse fibroblast NIH3T3 cell line, activated Rac1 and Cdc42 promoted the inactivation of Rb to allow E2F-mediated transcription, thus permitting the cell cycle progression from G1 into the S phase [30, 55–57].

Rho GTPase activity also affects cell cycle progression or inhibition via the activation of NFKB-dependent gene expression. NFKB activation by the Rac1 protein occurs when Rac binds to p67 (phox) to increase the activation of the NADPH oxidase system and the production of reactive oxygen species (ROS) [33, 56].

In GBM, Rac1 is a key contributor to cell survival, most likely via multiple signalling pathways [27]. For example, in an analysis of a set of erlotinib-resistant GBM cell lines in an expression analysis of 244 prospectively selected genes, Rac1 expression was shown to associate significantly with erlotinib-resistant glioblastoma. Erlotinib is a small molecule of tyrosine-kinase inhibitor that targets the EGFR. It has been studied as a targeted therapeutic strategy to take advantage of EGFR overexpression and its subsequent downstream (Figure 1) in GBM. While experimental GBM analysis showed favourable results; however, six clinical trials failed to prove any significant benefit, suggesting that different associated signalling pathways might regulate the proliferation of GBM. Therefore, interference with this Rac1 gene might enhance the proliferation inhibition of erlotinib against glioblastoma [27, 58].

Another study, using a Rac1 inhibitor in a retinoblastoma-deficient breast cancer cell line, demonstrated that Rac1 suppression leads to apoptosis [59]. This observation is consistent with the findings of an earlier study, in which the suppression of Rac1 led to glioma inhibition [54].

In addition to proliferation signalling, Rac1 is known as a key regulator of cell migration and invasion. This concept was proven by Chan et al. [60], who showed that the depletion of Rac1 in SNB19 and U87 glioblastoma cells lines strongly inhibited lamellipodia formation and cell migration.

**Figure 2:**
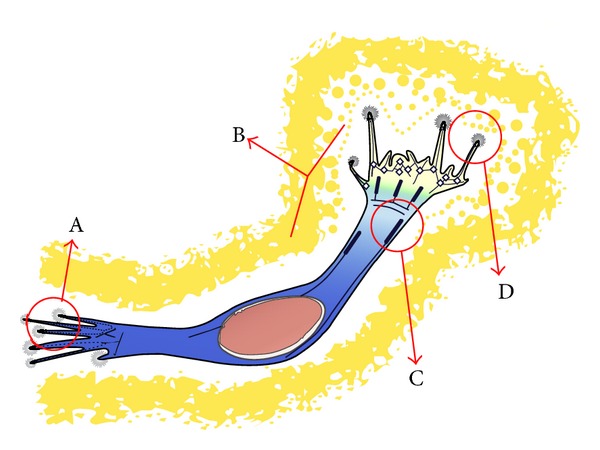
Schematic illustration of GBM cell. (A) Focal adhesion and (B) pseudopodium that is regulated by Rho GTPases to modulate actin cytoskeleton activity; (C) focal adhesions and actin cytoskeleton that support cell morphology and anchorage of the cell; (D) filopodia-ECM interactions that modulate actin-driven protrusions (adapted from O'Neill et al., 2010 [9]).

During migration, the actin fibers of the cell become polarised to form membrane protrusions through sheet-like extensions, such as pseudopodia, lamellipodia, filopodia, and invadopodia (Figure 2), which extend from the edges of the cells. These protrusions involve several signalling proteins that regulate filamentous actin and numerous structural membranes. The establishment of membrane anchors allows cytoskeletal contraction symphony, which finally moves the cell forward [17, 61]. Rac1 has been shown to localise at the leading edge of the moving cell, where it is activated by integrin-mediated cell adhesion and growth factors [9]. The role of Rac1 in cell migration is mediated through the formation of lamellipodia via the reorganisation of the actin cytoskeleton to generate locomotive force [62].

Rac1 activity has also been implicated in the aggressive phenotype. The aberrant activation of Rac1 stimulates neoplastic cell invasion via the activation of matrix metalloproteinase (MMP). MMP-2 and MMP-9 are examples of MMPs known to be upregulated in gliomas [62].

Cellular focal adhesions are points of linkage among the extracellular matrix (ECM), transmembrane integrin receptors, and the internal actin cytoskeleton. The integrin receptors are heterodimeric transmembrane complexes [9]. During tumour development, changes in integrin receptor expression, intracellular control of integrin function, and signals perceived from integrin receptor ligand binding influence the cell's ability to interact with the environment, enabling metastatic cells to convert from a sessile, stationary phenotype to a migratory and invasive phenotype [9].

Via this activity of focal adhesion kinases and their subsequent downstream molecules, a signalling network is established that culminates in the activation of GTPase proteins, such as Rac1. In turn, this determines the dynamic state of the actin cytoskeleton that is essential to the morphological progression of cell migration and adhesion [9].

Thus, Rac1 has been found to be involved in several pathways, explaining why it is so important in inducing a malignant phenotype. Several proteins act as effectors of Rac1 or are downstream of this gene region, including the p21-activated kinases (PAKs). For example, PAK1 is targeted by Rac1 to phosphorylate and activate the LIM kinase (LIMK), which phosphorylates cofilin. Cofilin phosphorylation triggers actin depolymerisation, resulting in the alteration of the cell structure [33].

The Rac1-associated activation of the actin-related protein-2/3 (ARP2/3) complex also activates actin polymerisation in lamellipodia. This polymerisation is triggered via Rac1 signalling, which binds to the WASP family verprolin homology domain-containing protein (WAVE) complex to release active WAVE and subsequently activates ARP2/3 [33, 57].

Other downstream targets of Rac1 are IQ motif containing GTPase activating protein-1 (IQGAP1), partner of Rac1 (POR1), plenty of SH3s (POSH), and CDC42-binding protein kinase alpha (CDC42BPA). To affect microtubule orientation and cell-to-cell adhesion, Rac1 binds to the actin-binding protein IQGAP1. The binding of IQGAP1 to the microtubule tip protein Clip170 captures growing microtubules at the leading edge of migrating fibroblasts, which results in cell polarisation [33, 63].

Therefore, Rac1 is essential for normal cell function; however, when improperly activated, it contributes to tumour cell growth, invasion, and angiogenesis [59]. Based on the culmination of evidence, the treatment of high-grade glioma should focus on targeting Rac1 [9].

## 7. NDV-Rac1 Interaction for Proliferation and Invasion Inhibition of GBM

A recent study reported that NDV is preferentially replicated in Rac1-activated cells [36], and a growing list of studies have directly or indirectly pointed to the Rac1 protein as a key factor in NDV infection of cancerous cells [7, 10]. This direction mainly discriminates the proliferation and invasive behaviour of normal and cancerous cells that are regulated by Rac1 protein signalling [33].

The involvement of Rac1 in NDV infection of GBM is the focus of this subtopic. To begin, the direct involvement of Rac1 in NDV-GBM cell tropism is discussed via two platforms: endocytosis viral entry and NDV-induced cell-to-cell fusion, called syncytium formation.

The paramyxovirus family, including NDV, primarily gain their entry into the infected cell when the HN viral protein recognises and binds cellular receptors at the plasma membrane, after which F protein triggers the merging of the viral envelope and plasma membrane, driving the introduction of the viral nucleocapsid into the cell. However, in a high viral concentration, NDV can enter the infected cell via caveolae-mediated endocytosis [64].

Caveolae are small, flask-shaped invaginations in the plasma membrane that contain high levels of cholesterol and glycosphingolipids as well as caveolins, structural proteins that form the caveolae [64]. Endocytosis is a cellular absorption process of large molecules that is primarily used for the nonselective internalisation of fluid and protein into the cell. The mechanism also drives the uptake of foreign particles, including viruses. Caveolae-mediated endocytosis has a strong connection with the actin cytoskeleton and involves the cholesterol-rich lipid raft domains at the plasma membrane [65], as well as a complex signalling pathway involving tyrosine kinases and phosphatases [66].

**Figure 3:**
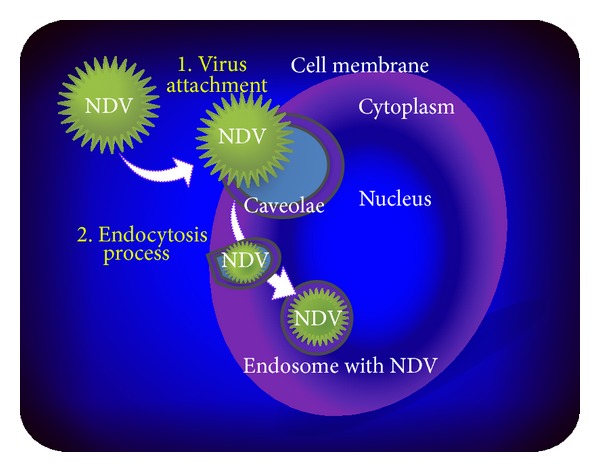
Schematic illustration of viral internalisation via caveolae-mediated endocytosis. Viral particles enter the cell through an endocytic pathway after viral-cell membrane fusion in a caveolae pocket that finally carries the virus into the cell endosome.

Therefore, some viruses are potentially contained within small invaginations in the plasma membranes of host cell that form the caveosome, which delivers virus particles to early endosomes (Figure 3) within the infected cells [66]. Cantín et al. [64] described the colocalization of NDV with caveolin and with the early endosome marker EEA1, leading to the suggestion that a certain percentage of the virus manages to penetrate the cell through caveolin-dependent endocytic pathways [67]. In that particular study, after 30 minutes of NDV infection, a strong colocalization of NDV HN protein and EEA1 was found, thus confirming that HN is targeted to early endosomes. EEA1 is an early endosomal antigen 1 marker protein used for localization of the virus in the intracellular structures. Endocytosis in paramyxovirus suggests that Rho GTPase Rac1 protein signalling has a role in the initial steps in the viral life cycle [68]. This suggestion is corroborated by a study on dynamic Rac1 and caveolin interaction that reported a direct interconnection of Rac1 as upstream of caveolin, where Rac1 activity promotes caveolin accumulation at Rac1-positive peripheral adhesions of the cell [69]. Thus, it seems probable that Rac1 activity interacts with caveolar regulation.

Remarkably, Rac1 protein is also involved in the phospholipase-D (PLD) regulation of phosphatidylcholine hydrolysis to yield phosphatidic acid and choline. Phosphatidic acid is a subsequent messenger involved in membrane remodelling events that are critical to cell growth, as well as vesicle trafficking into the cell and secretion [60, 70].

**Figure 4:**
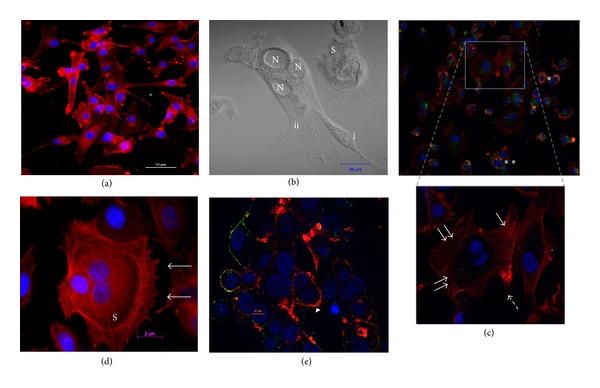
(a) Singular cells of untreated GBM with actin cytoskeleton staining (red colour). (b) Phase contrast microphotograph of NDV-infected GBM cells; (i) single-cell fusion process into the syncytium cells (ii) that is characterised by multiple nuclei (N). (c) Actin cytoskeleton staining of syncytium process in infected cells, showing actin cytoskeleton reorganisation (arrows in higher magnification microphotograph). (d) Completed actin reorganisation of three cells to become one syncytium cell (S). The asterisk (∗) and apoptotic syncytium (arrow head) in (c) and (e) represent actin cytoskeleton denaturation and cell death in a singular cell and a syncytium cell, respectively. The nucleus is stained blue and the NDV is stained green.

Furthermore, NDV infection is known to induce syncytium formation as a result of cell-to-cell fusion [10]. Our screening via live cell imaging of the GBM cell line showed that uninfected cells exhibit a migratory behaviour without intercell aggregation. In contrast, most migration GBM cell lines commonly fused with each other to form a giant syncytium cell with multiple nuclei (Figure 4(d)) after being treated with lentogenic NDV strain V4UPM, compared to untreated cells (Figure 4(a)). The syncytium cell also displayed the characteristic of actin reorganisation to form new borders surrounding the multiple nuclei, as indicated in Figures 4(b), 4(c), and 4(d).

According to Mansour et al. [10], enhanced fusogenicity has been shown to improve the oncolytic activity of NDV and vesicular stomatitis virus (VSV). In NDV-infected cells, syncytia are formed by the accumulation of newly synthesised viral HN and F glycoproteins, causing fusion with neighbouring cells. Thus, it can be postulated that apoptosis resistance may delay the apoptosis of NDV-infected cells, allowing fusion with an increased number of neighbouring cells and enhanced syncytium formation. As a benefit, the process helps to prolong the survival of cancer cells and allows the virus to replicate freely in the absence of an antiviral response [10].

Despite the extensive data [62, 71] regarding the mechanism of glioma cell migration, there is little information on the mechanism of cell-to-cell fusion. Taylor et al. [61] reported that in order to establish infection and promote cell fusion, the physical barrier imposed by the cortical actin meshwork in infected cells must be overcome. This process often requires the reprogramming of the actin cytoskeleton, thus explaining the reorganisation of the actin cytoskeleton network of NDV-infected glioma cells (Figures 4(c) and 4(d)).

NDV budding-out from the infected cell occurs with the involvement of a lipid raft on the cell membrane. Membrane lipid rafts are defined as cholesterol- and sphingolipid-rich microdomains in the exoplasmic leaflet of the cellular plasma membrane [72]. Lipid rafts associated with the actin cytoskeleton are thought to be sites of viral protein assembly in paramyxovirus budding-out and are released from the infected cell upon replication of the virus. This notion was first proposed after the detection of large quantities of actin in purified preparations of paramyxoviruses, including NDV [73].

The involvement of NDV in the regulation of or interaction with the cellular actin cytoskeleton has been crucial in its establishment of infection. This proposition is supported by a study that showed that cells infected by other paramyxoviruses, such as Hendra virus and simian virus 5, often display actin reorganisation, which suggests that Rac1 has a role in the early steps of the viral life cycle [68].

Thus, the role of Rac1 as a pleiotropic regulator of multiple cellular functions, including actin cytoskeletal reorganisation, gene transcription, and cell migration [74], needs to be elucidated further to explain the mechanism of the NDV-Rac1 interaction in human cancer cells. Puhlmann et al. [36] showed that Rac1 overexpression led to a significant increase in NDV replication in the cell pool, accompanied by increased oncolysis, thus identifying Rac1 as an oncogenic protein that is essential for NDV sensitisation and replication in tumorigenic cells.

In glioma cells, depletion of Rac1 expression by siRNA strongly inhibits lamellipodia formation and results in a decrease in cell migration and invasion. Moreover, inhibition of Rac1 activity via a dominant negative form of Rac1 induces apoptosis in primary and glioma cell lines, but not in normal adult astrocytes [14].

This is particularly interesting, as our experience with live cell imaging also showed the repressed mobility of infected cells at approximately 12 hours after infection. In the live cell movie, initial recording showed active cellular migration of the cells all over the microscopic view in both untreated and NDV V4UPM-treated GBM cells. However, the cellular migration was repressed as both singular and syncytium infected cells appeared to struggle locally and finally underwent cytolysis (video supplement). In contrast, the mobility behaviour continued in the untreated cells.

Ultimately, both singular and syncytium NDV-infected cells undergo apoptosis as showed in bottom-left quadrant of the video and Figure 4(e) [4, 75]. However, the live cell video supplement also shows that the syncytium formation induced temporary death resistance compared to the singular infected cells, indicating that NDV possibly infects glioma cells and exploits the cellular cytoskeleton for cell fusion to extend the infected cell's survival time and allow its replication.

Therefore, the fact that NDV replication requires Rac1 for tropism in human cancer cells [36], as well as the role of Rac1 in cell migration [60] and actin reorganisation [76], the rearrangement of actin cytoskeletons in syncytium cells (Figure 4(d)) and the repressed mobility of NDV-treated GBM cells observed in the live cell video have placed Rac1 in NDV tropism in GBM. In our previous work [7], Rac1 gene expression in NDV-treated GBM at 24-hour intervals showed significant Rac1 gene downregulation. Guided by the acute cytolytic effects observed in live cells, Rac1 protein expression was screened at 3, 6, 9, and 12 hours. The results indicated that the Rac1 protein was linearly upregulated at 3, 6, and 9 hours after infection, followed by significant downregulation at 12 hours after infection.

Ibrahim [77] reported that lentogenic NDV strain V4UPM infection of a GBM cell line induced cell cycle arrest at the S phase. In breast cancer cell lines, siRNA treatment against Rac1 suppressed the protein and its downstream NFKB, leading to S phase cell cycle arrest and apoptosis [59]. These interactions have indirectly placed the NDV interaction in the proliferation and invasion of the GBM cell via Rac1 protein.

## 8. NDV-Induced Apoptosis Pathways

**Figure 5:**
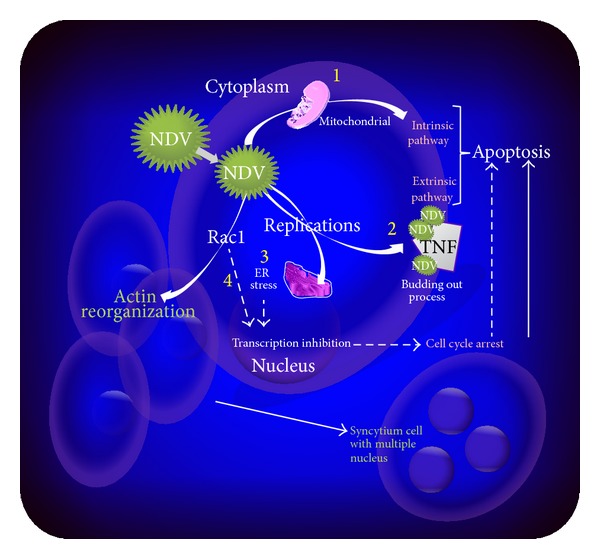
Multiple signalling reported in NDV induces cell death pathways in a cancerous cell. NDV infection of cancerous cells potentially induces direct apoptosis via intrinsic (1) or extrinsic (2) pathways. The NDV replication activity also induces ER stress (3), which triggers the transcription inhibition that leads to cell cycle arrest. NDV interactions with Rac1 (4) protein to induce syncytium formation also potentially induces cell cycle arrest, while cellular actin reorganisation in syncytium cells also induces denaturing of the actin cytoskeleton, which leads to cell death.

The NDV-Rac1 interaction is not the only mechanism, as many other pathways have been discovered.  Figure 5 summarises the NDV-GBM interactions that lead to cell death. Elankumaran et al. [4] reported that NDV primarily initiates apoptosis via the intrinsic pathway. NDV infection induces mitochondrial permeability, leading to the release of cytochrome C. It further binds to procaspase 9 to form an apoptosome, which further activates Caspase-9 and Caspase-3, subsequently leading to apoptosis [4, 75].

NDV infections of cancerous cells also induce cell death via extrinsic apoptosis signalling, rather than via an intrinsic pathway. The pathway is triggered by death ligands such as tumours necrosis factor (TNF), which induces TNF-related apoptosis-inducing ligand (TRAIL) and subsequently promotes cell death via Caspase-8. Proteolytic Caspase-8 cleaves and activates executioner Caspase-3, leading to further cell death [4].

Knowledge of asynchronous apoptosis signalling between the intrinsic and extrinsic pathways of cells infected by NDV is limited. The interference of NDV with the cellular actin cytoskeleton to sustain syncytium cell viability for their replication might be a cause. The NDV viral proteins line up on the actin cytoskeleton of infected cells for the budding-out to produce new virion progeny, which might suggest secondary apoptosis induction via the extrinsic pathway. Janmey [78] described that during apoptosis, major cytoskeleton filaments, including actin, cytokeratin, and microtubules, are degraded. The degradation of actin causes the cell to collapse and induces mechanical tension, cell detachment, and subsequent cell death.

## 9. Conclusion

In summary, a growing body of data has shown that the aberrant Rac1 oncogene is among the major regulators of GBM proliferation and invasion [58] and that NDV tropism in cancerous cells is connected with Rac1 protein signalling [36]. This finding is supported by the fact that cells infected with paramyxovirus often display actin reorganization, suggesting that Rac1 has a role in the early steps of the viral life cycle [68]. NDV has also been known to infect the GBM cell line and induce actin rearrangement in syncytium cells, leading to syncytium cell death. These findings indicate that lentogenic NDV is a promising bullet targeted at inhibiting GBM proliferation and invasion via its interaction with Rac1.
